# Angiogenesis and VEGF-expressing cells are identified predominantly in the fascia rather than in the muscle during the early phase of dermatomyositis

**DOI:** 10.1186/s13075-017-1481-z

**Published:** 2017-12-08

**Authors:** Ken Yoshida, Haruyasu Ito, Kazuhiro Furuya, Taro Ukichi, Kentaro Noda, Daitaro Kurosaka

**Affiliations:** Division of Rheumatology, Department of Internal Medicine, The Jikei University School of Medicine, 3-25-8 Nishi-shimbashi, Minato-ku, Tokyo, 105-8461 Japan

**Keywords:** Angiogenesis, Vascular endothelial growth factor, Fasciitis, Fascia, Muscle, Dermatomyositis, Polymyositis, Tumor necrosis factor-α

## Abstract

**Background:**

We previously demonstrated that fasciitis is a common lesion in dermatomyositis (DM) and that DM-associated fasciitis is detectable, as the result of the increased vascularity in the fascia, by power Doppler ultrasonography. We aimed to investigate whether angiogenesis and vascular endothelial growth factor (VEGF)-expressing cells in the fascia are histologically demonstrated during the early phase of DM, and whether inflammation is involved in angiogenesis and an increased number of VEGF-expressing cells.

**Methods:**

We prospectively evaluated 22 patients with DM and 11 patients with polymyositis (PM). Immunohistochemical staining for CD31, VEGF, and tumor necrosis factor-α (TNF-α) were performed on paraffin-embedded sections. The total vascular inflammation score (TVIS), angiogenesis score (AS), and numbers of VEGF-expressing and TNF-α-expressing cells were analyzed in the fascia and muscle.

**Results:**

Significant fasciitis was detected in most of the patients DM with or without myositis-specific/associated antibodies, while mild fasciitis was detected in four patients with PM, two of whom were positive for anti-aminoacyl-tRNA synthetase (anti-ARS) antibodies. The AS and the number of VEGF-expressing cells in the fascia of patients with DM were significantly greater than those of patients with PM; no significant difference was observed in muscle in patients with DM and PM. The number of VEGF-expressing cells in the fascia correlated with the AS of DM patients. In early-phase DM, the AS, the number of VEGF-expressing cells, and the TVIS in the fascia were significantly higher than in muscle. However, no significant differences were observed in these scores excluding the TVIS between muscle and the fascia in late-phase DM. In DM patients, the TVIS correlated with the AS in the fascia, while the number of TNF-α-expressing cells correlated with the TVIS and the number of VEGF-expressing cells in the fascia.

**Conclusion:**

Angiogenesis, the number of VEGF-expressing cells, and the degree of inflammation were higher in the fascia in DM than in PM, and were increased predominantly in the fascia rather than in the muscle in early-phase DM. The degree of inflammation correlated with that of angiogenesis in the fascia of DM. The fascia can therefore be a primary site of inflammation and angiogenesis in the pathogenesis of DM.

## Background

Dermatomyositis (DM) and polymyositis (PM) are characterized by idiopathic inflammatory myopathy (IIM) with and without characteristic cutaneous findings, respectively [1]. Both are heterogeneous autoimmune disorders, the pathogeneses of which remain unclear. The perivascular areas and the interfascicular septa in the muscle are typically considered to be the primary inflammatory sites in DM, while the endomysium of the muscle fibers within the fascicles are considered to be the primary inflammatory sites in PM [2, 3]. We previously provided histological evidence to show that fasciitis, which is a less frequently occurring and milder complication in PM, is a common lesion in early-phase DM (< 2 months) after the onset of muscle symptoms [4, 5]. Thus, the fascia is a crucial site for the initiation of inflammatory cell infiltration in DM. We recently reported that DM-associated fasciitis is detectable by power Doppler ultrasonography (PDUS) due to the increased vascularity in the fascia in patients with DM [6].

Some researchers have investigated whether the proliferation of capillaries is present in the muscle tissue of patients with IIM [7, 8]. Although the expression of an angiogenic factor, vascular endothelial growth factor (VEGF), was increased in muscle in patients with DM and PM in comparison to normal healthy controls, there were no differences between the DM and PM groups in VEGF expression, or among the three groups with regard to the number of capillaries in the muscle [7]. Intensive investigations of muscle tissue were undertaken to elucidate the pathogenesis of IIM; however, the fascia remained largely unexplored in patients with IIM. We therefore focused on the fascia in patients with DM and PM, especially those with DM, and undertook this study to examine whether angiogenesis and the number of VEGF-expressing cells were predominantly detected in the fascia rather than the muscle during the early phase of DM.

## Methods

### Patients

The study population included 54 patients who were newly diagnosed with definite or probable DM or PM according to the Bohan and Peter criteria, between June 2006 and January 2011 and between October 2013 and June 2015 at the Division of Rheumatology of Jikei University Hospital in Tokyo, Japan. Among these 54 patients, 33 patients pretreatment, consisting of 22 with DM (male, n = 10; female, n = 12; mean age, 55.3 years; range 30–73 years) and 11 with PM (male, n = 5; female, n = 6; mean age, 65.1 years; range 54–84 years), who received *en bloc* biopsy, were prospectively analyzed in the present study. Among the 22 patients with DM, 13 patients had interstitial lung disease (ILD), 5 patients had cancer, and 8 patients had myositis-specific/associated antibodies such as anti-aminoacyl-tRNA synthetase (anti-ARS) antibodies including anti-Jo-1 antibodies, anti-Mi-2 antibodies, anti-MDA-5 antibodies, anti-SRP antibodies, or anti-Ku antibodies. In contrast, among the 11 patients with PM, 3 patients had ILD, 1 patient had cancer, and 4 patients had myositis-specific/associated antibodies. The mean levels of serum creatine kinase (CK) in patients with DM and PM were 1052.1 U/ml (range 35–6530 U/ml) and 1409.9 U/ml (range 156–4304 U/ml), respectively. The disease duration is defined as the period from the onset of muscle symptoms (including muscle pain, weakness, and a stretched feeling) at the site of the *en bloc* biopsy until biopsy. The mean disease duration in patients with DM and PM was 10 weeks (range 1–52 weeks) and 73.5 weeks (range 5–312 weeks), respectively (Table 1). We previously showed that histological fasciitis was less frequently detected in patients with PM than with DM [4–6]. Thus, in the present study, patients with PM were assigned to a comparison group. None of the PM patients had finger flexion weakness, which is a component in the European Neuromuscular Centre criteria for inclusion body myositis [9]. This study did not include patients who had been receiving immunosuppressive agents or prednisolone (> 10 mg/day) before *en bloc* muscle biopsy and those who had already gotten general muscle biopsy without the fascia. The study design was approved by the ethics committee of The Jikei University School of Medicine, and informed consent was obtained from all of the patients.

**Table 1:** The clinical characteristics of the patients DM (n = 22) and PM (n = 11)

Number	Diagnosis	ILD	Cancer	CK	Jo1	ARS	Other^a^	Fasciitis^b^	Duration^c^	Biopsy site
DM										
1	Definite	No	Yes	249	N	NA	NA	+	1 week	Sartorius
2	Definite	Yes	Yes	303	N	NA	NA	+/–	2 weeks	Biceps brachii
3	Definite	Yes	No	1491	N	N	Ku	+	2 weeks	Biceps brachii
4	Definite	Yes	No	470	N	N	MDA5	+/–	2 weeks	Deltoid
5	Probable	Yes	No	480	N	NA	NA	+/–	3 weeks	Deltoid
6	Definite	Yes	No	176	N	N	N	+/–	3 weeks	Deltoid
7	Definite	Yes	No	1823	N	N	N	+/–	4 weeks	Biceps brachii
8	Definite	No	No	363	N	N	N	+	4 weeks	Rectus femoris
9	Probable	Yes	No	229	N	NA	NA	+	5 weeks	Biceps brachii
10	Definite	Yes	No	2833	P	P	NA	+	6 weeks	Biceps brachii
11	Probable	Yes	No	751	P	NA	NA	+	6 weeks	Biceps brachii
12	Definite	No	No	77	N	NA	NA	+	7 weeks	Biceps brachii
13	Definite	Yes	Yes	246	N	N	N	+	2 months	Triceps brachii
14	Definite	No	No	6530	N	N	Mi-2	+	2 months	Biceps brachii
15	Definite	No	No	895	N	N	Mi-2	+	2 months	Biceps brachii
16	Definite	No	Yes	246	N	NA	NA	+	3 months	Biceps brachii
17	Definite	No	Yes	1342	N	NA	NA	+	3 months	Deltoid
18	Probable	Yes	No	2428	P	P	N	+	4 months	Biceps brachii
19	Definite	Yes	No	35	N	NA	NA	+	4 months	Deltoid
20	Definite	No	No	415	N	N	Mi-2	+	5 months	Biceps brachii
21	Definite	Yes	No	1683	N	P	N	+	6 months	Biceps brachii
22	Definite	No	No	82	N	N	N	+	1 year	Sartorius
PM										
23	Probable	Yes	No	615	N	N	NA	+/–	5 weeks	Pectoralis major
24	Probable	No	No	1387	N	N	NA	–	3 months	Vastus medialis
25	Probable	No	No	1681	N	N	NA	–	3.5 months	Triceps brachii
26	Probable	Yes	No	1691	N	N	SRP	–	5 months	Semimembranous
27	Probable	No	No	4304	N	N	NA	–	6 months	Triceps brachii
28	Probable	No	No	1323	N	N	NA	–	9 months	Biceps brachii
29	Probable	No	Yes	2029	N	NA	NA	–	1 year	Sartorius
30	Probable	No	No	156	N	P	NA	+/–	2 years	Vastus lateralis
31	Definite	No	No	486	P	NA	NA	–	3 years	Vastus medialis
32	Definite	No	No	1583	N	N	N	+/–	6 years	Vastus lateralis
33	Probable	Yes	No	254	N	P	N	+/–	NA	Semimembranous

*DM* dermatomyositis, *PM* polymyositis, *ILD* interstitial lung disease, *CK* creatine kinase, *Jo1* anti-Jo-1 antibodies, *ARS* anti-ARS antibodies including anti-Jo-1 antibodies detected by EUROLINE or MESACUP^TM^, *N* negative, *P* positive, *NA* not available

^a^Other myositis-specific/associated antibodies detected by EUROLINE or MESACUP^TM^ (anti-Mi-2, anti-MDA5, anti-SRP, or anti-Ku antibodies), Ku anti-Ku antibodies, MDA5 anti-melanoma differentiation-associated gene 5 antibodies, Mi-2 anti-Mi-2 antibodies, SRP anti-signal recognition particle antibodies

^b^Plus symbol (+) represents histological significant fasciitis (TVIS ≥ 3), Plus or minus symbol (+/–) represents histological mild fasciitis (0 < TVIS < 3), minus symbol (–) represents no inflammatory cell infiltration in the fascia (TVIS = 0)

^c^Duration is the period from the onset of muscle symptoms at the site of *en bloc* biopsy up to the biopsy

### *En bloc* biopsy

For all patients, axial gadolinium-enhanced fat-suppressed T1-weighted (Gd-T1W) magnetic resonance (MR) images of the upper arms and femurs were obtained in addition to coronal and axial short tau inversion recovery (STIR) and axial T1-weighted MR images before *en bloc* biopsy. In *en bloc* biopsy, after marking the site where the patients were conscious of muscle symptoms (even mild muscle pain, weakness, or a stretched feeling) and/or where magnetic resonance imaging (MRI) showed abnormal findings, which included high signal intensity on STIR and abnormal enhancement on Gd-T1W as previously described, the skin, subcutaneous tissue, fascia, and muscle were resected to obtain a biopsy specimen of approximately 2 cm in size [4]. The biceps brachii muscle was selected as the first choice of the biopsy site. When muscle symptoms and abnormal MRI findings were not detected in the biceps brachii muscle, the biopsy was performed at another site with muscle symptoms and/or abnormal findings on MRI. When it was difficult to perform *en bloc* biopsy (e.g. at sites with thick subcutaneous tissue), a myofascial biopsy, in which only the fascia and muscle were resected, was performed.

### Immunohistochemical staining and histological analysis

All of the biopsy samples were fixed in 10% neutral-buffered formalin and embedded in paraffin. For each biopsy, 3-μm-thick paraffin-embedded sections were stained with hematoxylin and eosin (H&E) and Elastica van Gieson. The total vascular inflammation score (TVIS) was defined as the total number of aggregates of ≥ 50 perivascular inflammatory cells per 4-mm^2^ area of the fascia or muscle in the three fields with the most remarkable infiltration of perivascular inflammatory cells to quantify the degree of inflammation, as previously described [4]. In addition, our previous scoring system was modified as follows: the TVIS was defined as 1 if the total number of aggregates of 1–49 perivascular inflammatory cells per 4-mm^2^ area in three fields was ≥ 2. Immunohistochemical staining was performed on paraffin-embedded sections using mouse anti-human CD31 monoclonal antibodies (Dako, dilution rate 1:500), mouse anti-human VEGF monoclonal antibodies (Millipore, dilution rate 1:500), rabbit anti-human tumor necrosis factor-α (TNF-α) polyclonal antibodies (abcam, dilution rate 1:2500). The angiogenesis score (AS) was defined as the total number of CD31-expressing blood vessels with a recognizable luminal structure in the three high-power fields (× 200) that showed the most remarkable proliferation of the vessels in the fascia or muscle [6]. The numbers of VEGF-expressing and TNF-α-expressing cells were counted in the three high-power fields (× 400) that showed the largest accumulation of these cells in the fascia or muscle. The TVIS, AS, and the numbers of VEGF-expressing and TNF-α-expressing cells were counted by two investigators (KY and HI) who were blinded to the clinical data. These scores in the fascial and muscular areas were counted separately. The mean value of the two measurements was used for the statistical analysis, as previously described [4]. Histologically significant fasciitis was defined as a TVIS ≥3. Mild fasciitis was defined as a TVIS > 0 and < 3.

### Statistical analysis

The TVIS was compared between the fascial areas of the DM group and those of the PM group. The AS and number of VEGF-expressing cells in the DM group were compared with the respective scores in the PM group in the fascial and muscular areas. The early and late phases were defined as phases in which *en bloc* biopsy was performed earlier and later than 2 months after the appearance of muscle symptoms, respectively. The AS, number of VEGF-expressing cells, and TVIS in the fascial area were also compared with the respective scores in the muscular area in the patients with early-phase and late-phase DM. The number of TNF-α-expressing cells was compared between the DM and PM groups. The differences in the scores between two unpaired groups were analyzed using the Mann-Whitney *U* test, while the differences in the scores between two paired groups were analyzed using the Wilcoxon matched-pairs signed-rank test. The correlation between the scores was assessed by Spearman’s rank correlation coefficient. The statistical analyses were performed using the GraphPad Prism software program (version 4.0, GraphPad Software Inc., San Diego, CA, USA). The results are expressed as the mean ± SEM. *P* values < 0.05 were considered to indicate statistical significance.

## Results

### Angiogenesis and an increased number of VEGF-expressing cells in the fascia of patients with DM

**Fig. 1:**
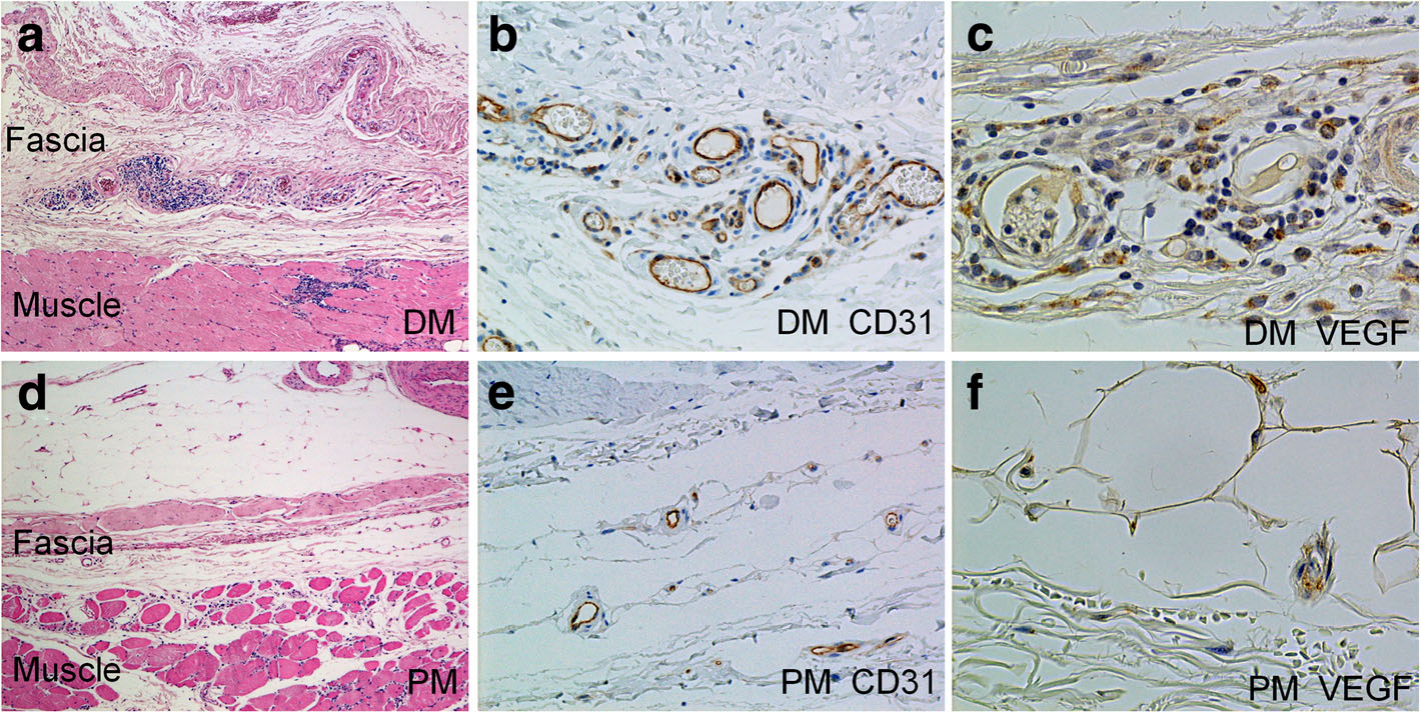
The histological images of fasciitis, CD31-expressing blood vessels and vascular endothelial growth factor (VEGF)-expressing cells in the fascia of patients with dermatomyositis (DM) or polymyositis (PM). **a** Fasciitis in a patient with DM. H&E staining shows massive inflammatory cell infiltration around the small blood vessels and hyperplasia of the fascia (original magnification × 50). **b** Immunohistochemical staining shows the proliferation of CD31-expressing capillaries and venules (brown) in the fascia of a patient with DM (original magnification × 200). **c** Immunohistochemical staining shows the accumulation of VEGF-expressing cells (brown) around the blood vessels in the fascia of a patient with DM (original magnification × 400). **d** No fasciitis is observed in a patient with PM (original magnification × 50). **e** Immunohistochemical staining for CD31 (brown) shows no angiogenesis in the fascia of a patient with PM (original magnification × 200). **f** Immunohistochemical staining shows a small number of VEGF-expressing cells (brown) in the fascia of a patient with PM (original magnification × 400)

**Fig. 2:**
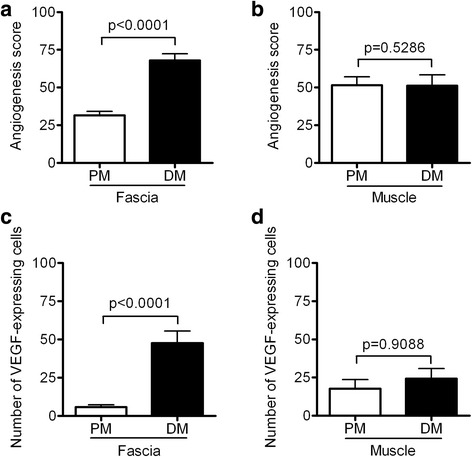
Comparison of the angiogenesis score (AS) and the number of vascular endothelial growth factor (VEGF)-expressing cells in the dermatomyositis (DM) group with the respective scores in the polymyositis (PM) group in the fascia and muscle. **a** The AS in the fascia of patients with DM or PM. **b** The AS in the muscle of patients with DM or PM. **c** The number of VEGF-expressing cells in the fascia of patients with DM or PM. **d** The number of VEGF-expressing cells in the muscle of patients with DM or PM. The results are expressed as the mean ± SEM. *P* values < 0.05 were considered to indicate statistical significance

Angiogenesis and the number of VEGF-expressing cells were separately evaluated in the fascia and the muscle. Elastica van Gieson (data not shown), H&E, and immunohistochemical staining for CD31 showed the abnormal proliferation of capillaries and venules, but not arteries, in the fascia of the DM group, while abnormal proliferation was rarely observed in the fascia of the PM group (Fig. 1a, b, d and e). In the DM group, the proliferation of capillaries and venules was predominantly observed in the fascia rather than the muscle. The AS in the fascia in the DM group (mean ± SEM, 67.9 ± 4.5) was significantly higher compared with that in the PM group (mean ± SEM, 31.5 ± 2.6) (*p* < 0.0001) (Fig. 2a). Conversely, the AS in the muscle of the DM (mean ± SEM, 51.3 ± 7.0) and PM groups (mean ± SEM, 51.6 ± 5.4) did not differ to a statistically significant extent (*p* = 0.5286) (Fig. 2b). However, the AS in the muscle of DM patients who had no or mild inflammatory cell infiltration in the muscle (TVIS in the muscle < 3) was lower than that of all 11 patients with PM (data not shown). Immunohistochemical staining showed that VEGF was mainly expressed on inflammatory mononuclear cells and slightly expressed on endothelial cells (Fig. 1c and f). The number of VEGF-expressing cells in the fascia of the DM group (mean ± SEM, 47.7 ± 7.8) was also significantly greater compared with that of the PM group (mean ± SEM, 5.9 ± 1.3) (*p* < 0.0001); whereas the number of VEGF-expressing cells in the muscle of the DM (mean ± SEM, 24.3 ± 6.7) and PM (mean ± SEM, 17.8 ± 5.9) groups did not differ to a statistically significant extent (*p* = 0.9088) (Fig. 2c and d). Although the disease duration in the PM group was significantly longer than that in the DM group, the disease duration in the PM group was not correlated with any of the analyzed values (AS, number of VEGF-expression cells, number of TNF-α-expressing cells, or TVIS) (data not shown). These results suggest that the disease duration did not affect any of the score values in this PM group.

### Positive correlation between the number of VEGF-expressing cells and angiogenesis in patients with DM

**Fig. 3:**
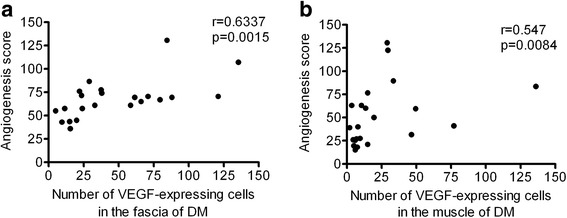
The relationships between angiogenesis and the number of vascular endothelial growth factor (VEGF)-expressing cells in patients with dermatomyositis (DM). **a** The positive correlation between the number of VEGF-expressing cells and the angiogenesis score (AS) in the fascia in patients with DM. **b** The positive correlation between the number of VEGF-expressing cells and the AS in muscle in patients with DM. *P* values < 0.05 were considered to indicate statistical significance

We next investigated the relationship between angiogenesis and one of the factors that most strongly induced angiogenesis (VEGF). VEGF-expressing cells and angiogenesis were primarily found in the fascia of the DM group. The number of VEGF-expressing cells was positively correlated with the AS of the fascia (*r* = 0.6337, *p* = 0.0015) and the muscle (*r* = 0.547, *p* = 0.0084) in the DM group (Fig. 3a and b). The number of VEGF-expressing cells in the muscle was also positively correlated with the AS in the PM group (data not shown).

### Angiogenesis, an increased number of VEGF-expressing cells, and inflammation in the fascia in patients with early-phase DM

**Fig. 4:**
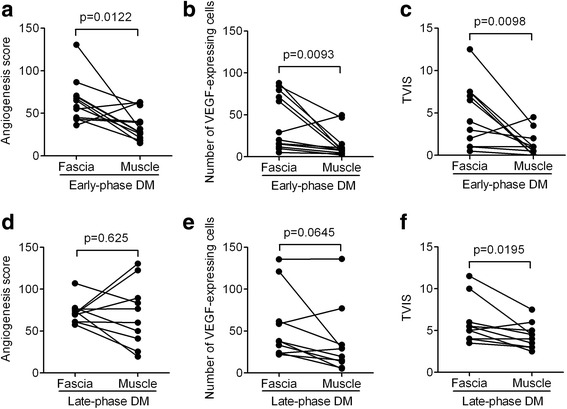
The comparison of the angiogenesis score (AS), number of vascular endothelial growth factor (VEGF)-expressing cells, and total vascular inflammation score (TVIS) in the fascia with the respective scores in muscle during the early and late phases of dermatomyositis (DM). **a** The AS in the muscle and fascia of patients with early-phase DM. **b** The number of VEGF-expressing cells in the muscle and fascia in patients with early-phase DM. **c** The TVIS in the muscle and fascia of patients with early-phase DM. **d** The AS in the muscle and fascia of patients with late-phase DM. **e** The number of VEGF-expressing cells in the muscle and fascia of patients with late-phase DM. **f** The TVIS in the muscle and fascia of patients with late-phase DM. *P* values < 0.05 were considered to indicate statistical significance

The AS, number of VEGF-expressing cells, and TVIS in the fascia in the DM group were compared with the respective scores in muscle in the early and the late phases after the onset of muscle symptoms. There were 12 patients with early-phase DM and 10 patients with late-phase DM. In early-phase DM, the AS in the fascia (mean ± SEM, 64.1 ± 7.4) was significantly higher compared with that in muscle (mean ± SEM, 35.9 ± 5.0) (*p* = 0.0122); in contrast, in late-phase DM, the AS in the fascia (mean ± SEM, 72.6 ± 4.4) and in muscle (mean ± SEM, 69.9 ± 12.0) did not differ to a statistically significant extent (*p* = 0.625) (Fig. 4a and d). Similarly, in early-phase DM, the number of VEGF-expressing cells in the fascia (mean ± SEM, 41.2 ± 3.2) was significantly greater compared with that in muscle (mean ± SEM, 14.1 ± 4.7) (*p* = 0.0093). In contrast, in late-phase DM, the number of VEGF-expressing cells in the fascia (mean ± SEM, 55.5 ± 12.9) and in muscle (mean ± SEM, 36.4 ± 12.8) did not differ to a statistically significant extent (*p* = 0.0645) (Fig. 4b and e). The TVIS in the fascia (mean ± SEM, 4.5 ± 1.1) was significantly higher in early-phase DM than that in muscle (mean ± SEM, 1.2 ± 0.4) (*p* = 0.0098), and also in late-phase DM, the TVIS in the fascia (mean ± SEM, 6.0 ± 0.8) was significantly higher than that in muscle (mean ± SEM, 4.4 ± 0.5) (*p* = 0.0195) (Fig. 4c and f).

### Positive correlation between inflammation and angiogenesis in the fascia of patients with DM

**Fig. 5:**
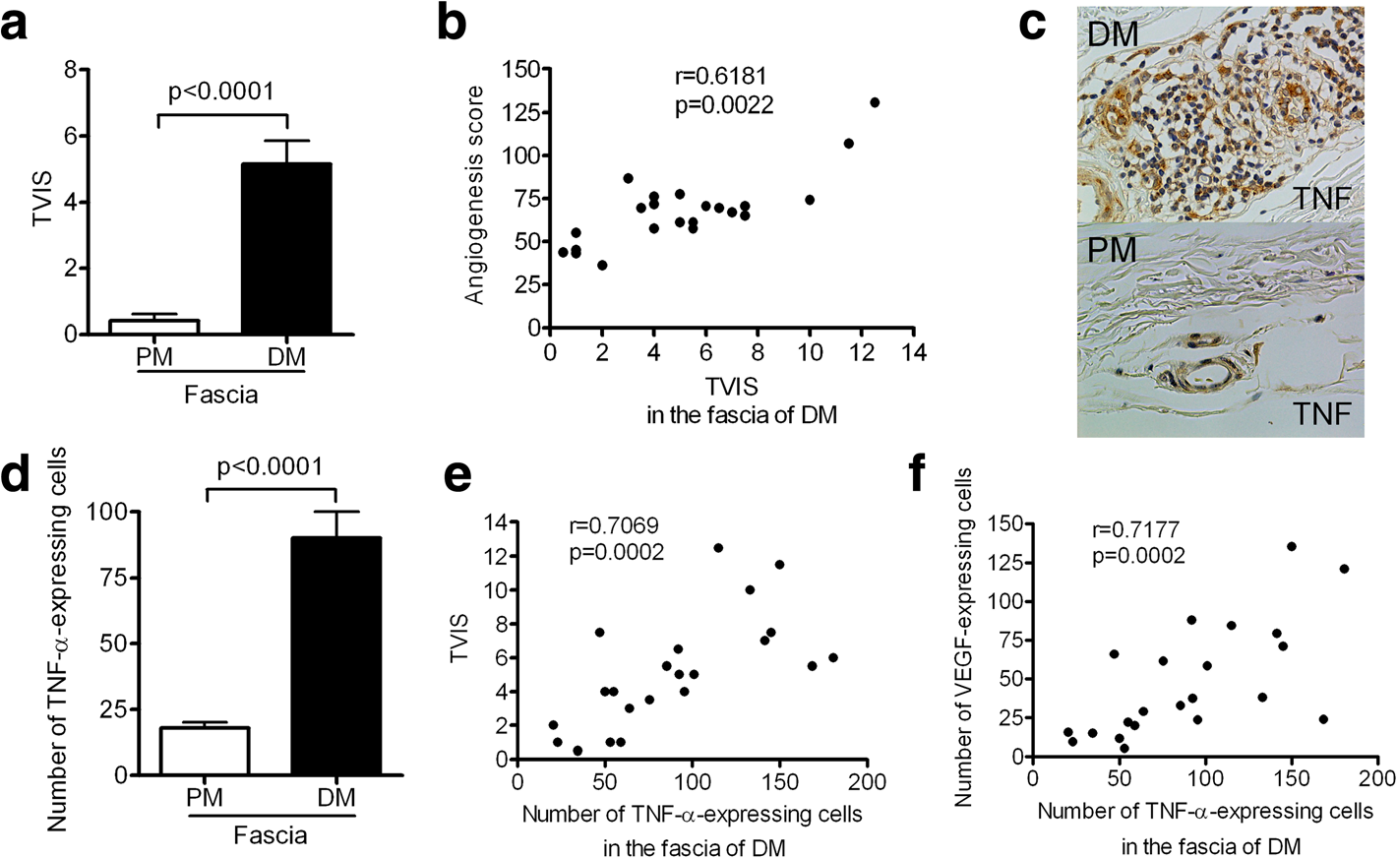
The relationship between inflammation and angiogenesis in the fascia in patients with dermatomyositis (DM). **a** The total vascular inflammation score (TVIS) of the fascia of patients with polymyositis (PM) or DM. **b** The positive correlation between the TVIS and AS in the fascia in patients with DM. **c** Immunohistochemical staining of the fascia for TNF-α (brown) in patients with DM or PM (original magnification × 400). **d** The number of TNF-α-expressing cells in the fascia of patients with PM or DM. **e** The number of TNF-α-expressing cells in the fascia of patients with DM was positively correlated with the TVIS **f** The number of TNF-α-expressing cells in the fascia of patients with DM was positively correlated with the number of VEGF-expressing cells. The results are expressed as the mean ± SEM. *P* values < 0.05 were considered to indicate statistical significance

In the following analysis, we examined the relationship between angiogenesis and inflammation, especially the number of TNF-α-expressing cells in the fascia of patients with DM. Histologically significant fasciitis, which was defined by a TVIS ≥3, was detected in 17 of 22 patients with DM and none of the 11 patients with PM. Mild fasciitis, which was defined by a TVIS > 0 and < 3, was detected in 5 of the 22 patients with DM and 4 of the 11 patients with PM (Table 1). Fasciitis, including mild fasciitis, was histologically detected in all patients with or without myositis-specific/associated antibodies in the DM group, while mild fasciitis, which had perivascular inflammatory cell infiltration, was detected in four patients with PM, two of whom had anti-ARS antibodies (Table 1). Fasciitis was not observed in patients with PM in whom inflammatory infiltrates predominantly surrounded the endomysium within the fascicles, and were not present at perivascular sites or in the interfascicular septa. The TVIS in the fascia in the DM group (mean ± SEM, 5.2 ± 0.7) was significantly higher in comparison to the PM group (mean ± SEM, 0.4 ± 0.2) (*p* < 0.0001) (Fig. 5a). The proliferation of capillaries and venules and hyperplasia of the fascia was predominantly found in the area infiltrated by inflammatory cells (Fig. 1a). In the DM group, the TVIS was correlated with the AS in the fascia (*r* = 0.6181, *p* = 0.0022) (Fig. 5b).

TNF-α is a proinflammatory cytokine that has been shown to be a pivotal factor early in the inflammatory cascade and to play a key role in the pathogenesis of inflammation [10]. It is involved in various rheumatic diseases, including DM. Immunohistochemical staining showed that TNF-α was predominantly expressed on endothelial cells and inflammatory mononuclear cells in the fascia in the DM group (Fig. 5c). The number of TNF-α-expressing cells in the fascia in the DM group (mean ± SEM, 90.1 ± 10) was significantly higher compared with that in the PM group (mean ± SEM, 17.9 ± 2.1) (*p* < 0.0001) (Fig. 5d), while the number of TNF-α-expressing cells in muscle in the DM and PM groups did not differ to a statistically significant extent (data not shown). In the fascia of the DM group, the number of TNF-α-expressing cells was correlated with the TVIS (*r* = 0.7069, *p* = 0.0002) (Fig. 5e) and with the number of VEGF-expressing cells (*r* = 0.7177, *p* = 0.0002) (Fig. 5f).

## Discussion

We found angiogenesis in the fascia in patients with DM but not in patients with PM. Although some researchers have provided data on the number of blood vessels or neovascularization in the muscle of patients with DM and PM, they did not report the data on the fascia [7, 8, 11]. Grundtman et al. reported that there was no significant difference in the number of CD31-expressing capillaries in muscle among patients with PM and DM and healthy controls. Similarly, in the present study, there was also no significant difference in the number of CD31-expressing vessels in muscle in the DM and PM groups. However, our previous study [6] and our present study showed that the number of CD31-expressing blood vessels in the fascia in patients with DM was significantly greater than in patients with PM, regardless of the number of CD31-expressing blood vessels in muscle. This suggests that the pathogenesis of the fascia differs markedly between DM and PM.

In the present study, immunohistochemical staining showed that the number of VEGF-expressing cells in the fascia in patients with DM was greater compared with that in patients with PM. In contrast, the number of VEGF-expressing cells within muscle in patients in the PM and DM groups did not differ to a statistically significant extent. Grundtman et al. showed that the total protein expression of VEGF in muscle in patients with PM and DM was significantly increased in comparison to healthy control subjects. However, there was no difference in the expression of VEGF within muscle in patients in the PM and DM groups [7]. This finding is in line with our results. VEGF, which is a major mediator of pathological angiogenesis [12], has a predominant role in both tumor-induced and inflammation-induced angiogenesis [13]. In the present study, the number of VEGF-expressing cells in the fascia was positively correlated with the AS, suggesting that VEGF is a principal angiogenic factor in DM-associated fasciitis.

In the early-phase DM, increased AS and number of VEGF-expressing cells were found in the fascia rather than in muscle. Conversely, in the late phase, the AS and the number of VEGF-expressing cells in the fascia and muscle did not differ to a statistically significant extent. We previously showed the possibility that inflammatory cell infiltration progressed from the fascia into muscle in patients with DM [4]. Moreover, PDUS predominantly detected increased blood flow signals in the fascia rather than in muscle [6]. Our data suggest that the angiogenesis induced by VEGF may progress from the fascia into muscle in patients with DM. Any lack of findings of inflammation and angiogenesis histologically detected in the muscle tissue at a site with muscle symptoms, such as myalgia, may be due in part to DM-associated fasciitis in the early phase before the progression of inflammation and angiogenesis to muscle.

We previously showed that DM is frequently associated with fasciitis, whereas no such association is noted with PM [4–6]. In the present study, fasciitis, including mild fasciitis, was detected in all patients with DM regardless of the presence of myositis-specific/associated antibodies, suggesting that fasciitis results from the properties of DM. In contrast, mild fasciitis, which had perivascular inflammatory cell infiltration, was detected in four patients with PM, two of whom were positive for anti-ARS antibodies, but not in patients with PM in whom inflammatory infiltrates predominantly surrounded the endomysium within the fascicles. In general, inflammatory infiltrates are predominantly found at perivascular sites or in the interfascicular septa in DM, while they surround and invade non-necrotic muscle fibers expressing major histocompatibility class I antigens within the fascicles in PM [2, 3]. Noguchi et al. showed that in antisynthetase syndrome, inflammatory infiltrates were present at perimysial sites, normally perivascular sites, but that they did not surround the endomysium or invade into non-necrotic muscle fibers within the fascicles, as typically observed in PM [14]. Their study suggests that the sites of inflammatory cell infiltration in antisynthetase syndrome are similar to those in DM. We therefore believe that fasciitis can be frequently detected in anti-ARS antibody-positive patients without typical skin rash who are diagnosed with PM according to the Bohan and Peter criteria; however, fasciitis is rarely detected in patients who are diagnosed with PM based on the typical histological findings of PM.

In the patients with DM in the present study, angiogenesis was accompanied by inflammatory cell infiltration. The TVIS in the fascia in the DM group was significantly greater compared with the PM group, which included a larger number of patients than in our previous report. [4] The degree of inflammation (TVIS) and angiogenesis in the fascia were both significantly increased during early-phase DM compared with those in muscle. Furthermore, there was significant positive correlation between the TVIS and the AS in the fascia in patients with DM. Grundtman et al. also showed that the number of capillaries expressing CD31 in muscle was significantly higher in patients with PM/DM with inflammatory cell infiltrates before treatment than in those without inflammatory cell infiltrates. In chronic inflammatory disorders, hypoxia is associated with the accumulation of inflammatory cells and the overexpression of hypoxia-inducible factor, which promotes the transcription of several angiogenic genes, including VEGF, and consequently induces angiogenesis [15–18]. For instance, in patients with rheumatoid arthritis, hypoxia mainly occurs due to hyperplasia of the synovial lining and the augmented infiltration of immune cells [19–21]. In our study, the marked proliferation of blood vessels in the fascia and hyperplasia of the fascia were present at the sites of intense inflammatory cell infiltration in patients with DM. Local inflammatory processes are considered a potential source of angiogenesis in DM-associated fasciitis.

TNF-α is generally considered to be a proinflammatory cytokine that plays a principal role in initiating the cascade of activation of other cytokines and growth factors in the inflammatory response. VEGF is produced in response to stimulation by proinflammatory cytokines [22]. Although Kuru et al. and Lundberg et al. previously reported that TNF-α was expressed in muscle in patients both with PM and with DM, the expression levels in patients with PM and DM did not differ to a significant extent [23, 24]. In the present study, the number of TNF-α-expressing cells in the fascia in patients with DM was significantly higher than in patients with PM, whereas the number in muscle in patients with PM and DM did not differ to a statistically significant extent as they previously reported. Furthermore, the number of TNF-α-expressing cells was positively correlated with the TVIS and the number of VEGF-expressing cells in the fascia of patients with DM. TNF-α enhanced the expression of angiogenic ligands, such as VEGF, basic fibroblast growth factor (bFGF), and IL-8 [25]. Anti-VEGF antibodies inhibit TNF-α-induced angiogenesis in the rabbit cornea [25]. These reports and our data suggest that TNF-α is a potential angiogenesis factor in the fascia of patients with DM, which increases the expression of VEGF.

With regard to patients with IIM, there are conflicting reports about the responses to TNF-α blocking agents. Datmalchi et al. reported that infliximab treatment was not effective in treating refractory inflammatory myopathies in an open pilot study [26]. Brunasso et al. concluded in their systematic literature review that the use of the TNF-α blocking agents had the potential to trigger the onset of PM, DM, and antisynthetase syndrome in patients with chronic inflammatory diseases [27]. However, several case reports and case series have suggested the beneficial effects of TNF-α blocking agents such as infliximab, etanercept, and adalimumab in patients with DM or PM [28–32]. It appears that anti-TNF-α treatment could be of benefit to a subset of patients with IIM, especially those with early-stage DM with interstitial lung disease [30–32]. A further study would be needed to clarify the effects of anti-TNF-α therapy in the different subsets of DM.

The present study had the limitation of having a small study population. A further study of a larger cohort of patients with PM and DM is needed to clarify the findings.

## Conclusions

Angiogenesis, an increased number of VEGF-expressing cells, and intense inflammation were demonstrated predominantly in the fascia rather than in muscle in patients with DM during the early phase after the onset of muscle symptoms. The inflammation induced by TNF-α is involved in angiogenesis in the fascia of patients with DM. These data suggest that the fascia can be a primary site of inflammation and angiogenesis in the pathogenesis of DM.

## Abbreviations

ARS: Aminoacyl-tRNA synthetase
AS: Angiogenesis score
CK: Creatine kinase
DM: Dermatomyositis
Gd-T1W: Gadolinium-enhanced fat-suppressed T1-weighted
H&E: Hematoxylin and eosin
IIM: Idiopathic inflammatory myopathy
IL: Interleukin
ILD: Interstitial lung disease
MDA5: Melanoma differentiation-associated gene 5
MR: Magnetic resonance
PDUS: Power Doppler ultrasonography
PM: Polymyositis
SRP: Signal recognition particle
STIR: Short tau inversion recovery
TNF-α: Tumor necrosis factor-α
TVIS: Total vascular inflammation score
VEGF: Vascular endothelial growth factor

## References

[1] Bohan A, Peter JB (1975). Polymyositis and dermatomyositis (first of two parts). N Engl J Med..

[2] Dalakas MC (1995). Immunopathogenesis of inflammatory myopathies. Ann Neurol..

[3] Engel AG, Hohlfeld R, Banker BQ. The polymyositis and dermatomyositis syndrome. In: Engel AG, Franzini-Armstrong C (eds) Myology. McGraw-Hill, New York. 2006;1335–83.

[4] Yoshida K, Kurosaka D, Joh K (2010). Fasciitis as a common lesion of dermatomyositis, demonstrated early after disease onset by *en bloc* biopsy combined with magnetic resonance imaging. Arthritis Rheum..

[5] Noda K, Yoshida K, Ukichi T, Furuya K, Hirai K, Kingetsu I (2017). Myalgia in patients with dermatomyositis and polymyositis is attributable to fasciitis rather than myositis: a retrospective study of 32 patients who underwent histopathological examinations. J Rheumatol..

[6] Yoshida K, Nishioka M, Matsushima S, Joh K, Oto Y, Yoshiga M (2016). Brief report: power Doppler ultrasonography for detection of increased vascularity in the fascia: a potential early diagnostic tool in fasciitis of dermatomyositis. Arthritis Rheumatol..

[7] Grundtman C, Tham E, Ulfgren AK, Lundberg IE (2008). Vascular endothelial growth factor is highly expressed in muscle tissue of patients with polymyositis and patients with dermatomyositis. Arthritis Rheum..

[8] Nagaraju K, Rider LG, Fan C, Chen YW, Mitsak M, Rawat R (2006). Endothelial cell activation and neovascularization are prominent in dermatomyositis. J Autoimmune Dis..

[9] Lloyd TE, Mammen AL, Amato AA, Weiss MD, Needham M, Greenberg SA (2014). Evaluation and construction of diagnostic criteria for inclusion body myositis. Neurology..

[10] Harriman G, Harper LK, Schaible TF (1999). Summary of clinical trials in rheumatoid arthritis using infliximab, an anti-TNFalpha treatment. Ann Rheum Dis..

[11] Emslie-Smith AM, Engel AG (1990). Microvascular changes in early and advanced dermatomyositis: a quantitative study. Ann Neurol..

[12] Ferrara N, Carver-Moore K, Chen H, Dowd M, Lu L, O’Shea KS (1996). Heterozygous embryonic lethality induced by targeted inactivation of the VEGF gene. Nature..

[13] Carmeliet P, Jain RK (2000). Angiogenesis in cancer and other diseases. Nature..

[14] Noguchi E, Uruha A, Suzuki S, Hamanaka K, Ohnuki Y, Tsugawa J (2017). Skeletal muscle involvement in antisynthetase syndrome. JAMA Neurol..

[15] Ramakrishnan S, Anand V, Roy S (2014). Vascular endothelial growth factor signaling in hypoxia and inflammation. J Neuroimmune Pharmacol..

[16] Coussens LM, Werb Z (2002). Inflammation and cancer. Nature..

[17] Naldini A, Carraro F (2005). Role of inflammatory mediators in angiogenesis. Curr Drug Targets Inflamm Allergy..

[18] Konisti S, Kiriakidis S, Paleolog EM (2012). Hypoxia–a key regulator of angiogenesis and inflammation in rheumatoid arthritis. Nat Rev Rheumatol..

[19] Peters CL, Morris CJ, Mapp PI, Blake DR, Lewis CE, Winrow VR (2004). The transcription factors hypoxia-inducible factor 1alpha and Ets-1 colocalize in the hypoxic synovium of inflamed joints in adjuvant-induced arthritis. Arthritis Rheum..

[20] Lee YA, Kim JY, Hong SJ, Lee SH, Yoo MC, Kim KS (2007). Synovial proliferation differentially affects hypoxia in the joint cavities of rheumatoid arthritis and osteoarthritis patients. Clin Rheumatol..

[21] Jeon CH, Ahn JK, Chai JY, Kim HJ, Bae EK, Park SH (2008). Hypoxia appears at pre-arthritic stage and shows co-localization with early synovial inflammation in collagen induced arthritis. Clin Exp Rheumatol..

[22] Marrelli A, Cipriani P, Liakouli V, Carubbi F, Perricone C, Perricone R (2011). Angiogenesis in rheumatoid arthritis: a disease specific process or a common response to chronic inflammation?. Autoimmun Rev..

[23] Kuru S, Inukai A, Liang Y, Doyu M, Takano A, Sobue G (2000). Tumor necrosis factor-alpha expression in muscles of polymyositis and dermatomyositis. Acta Neuropathol..

[24] Lundberg I, Ulfgren AK, Nyberg P, Andersson U, Klareskog L (1997). Cytokine production in muscle tissue of patients with idiopathic inflammatory myopathies. Arthritis Rheum..

[25] Yoshida S, Ono M, Shono T, Izumi H, Ishibashi T, Suzuki H (1997). Involvement of interleukin-8, vascular endothelial growth factor, and basic fibroblast growth factor in tumor necrosis factor alpha-dependent angiogenesis. Mol Cell Biol..

[26] Dastmalchi M, Grundtman C, Alexanderson H, Mavragani CP, Einarsdottir H, Helmers SB (2008). A high incidence of disease flares in an open pilot study of infliximab in patients with refractory inflammatory myopathies. Ann Rheum Dis..

[27] Brunasso AM, Abererb W, Massone C (2014). New onset of dermatomyositis/polymyositis during anti-TNF-α therapies: a systematic literature review. Scientific World Journal..

[28] Hengstman GJ, van den Hoogen FH, Barrera P, Netea MG, Pieterse A, van de Putte LB (2003). Successful treatment of dermatomyositis and polymyositis with anti-tumor-necrosis-factor-alpha: preliminary observations. Eur Neurol..

[29] Efthimiou P, Schwartzman S, Kagen LJ (2006). Possible role for tumour necrosis factor inhibitors in the treatment of resistant dermatomyositis and polymyositis: a retrospective study of eight patients. Ann Rheum Dis..

[30] Dold S, Justiniano ME, Marquez J, Espinoza LR (2007). Treatment of early and refractory dermatomyositis with infliximab: a report of two cases. Clin Rheumatol..

[31] Park JK, Yoo HG, Ahn DS, Jeon HS, Yoo WH (2012). Successful treatment for conventional treatment-resistant dermatomyositis-associated interstitial lung disease with adalimumab. Rheumatol Int..

[32] Chen D, Wang XB, Zhou Y, Zhu XC (2013). Efficacy of infliximab in the treatment for dermatomyositis with acute interstitial pneumonia: a study of fourteen cases and literature review. Rheumatol Int..

